# The optimized jugular vein catheterization reinforced cocaine self-administration addictive model for adult male Sprague–Dawley rats

**DOI:** 10.1038/s41598-022-15833-z

**Published:** 2022-07-09

**Authors:** Yang Li, Liang Qu, Nan Li, Xin Wang, Ping Wang, Shun-nan Ge, Xue-lian Wang

**Affiliations:** 1 Department of Neurosurgery, Tangdu Hospital, Air Force Medical University, Xi’an, Shaanxi 710038 People’s Republic of China; 2 Department of Neurosurgery, General Hospital of Central Theater Command, Wuhan, Hubei 430070 China

**Keywords:** Biotechnology, Neuroscience, Developmental biology

## Abstract

The self-administration (SA) model represents one of the most important and classic methods for drug addiction, and jugular vein catheterization is one of the most critical techniques in this animal model. We aimed to explore an optimized scheme to improve the success rate of rat jugular vein catheterization and SA model. Our experiment provided an optimized scheme which including numerous details, materials, approaches, updated techniques and protocols. Our experimental group consisted of 120 adult male Sprague–Dawley rats, which were divided into the Traditional Operation group (TO group) and the Optimized Operation group (OO group) by the random number table method and then further individually divided into the Saline Training group and the Cocaine Training group for the following SA training. Our results showed that the success rate of the jugular vein catheterization in the OO group was significantly greater than that in the TO group (93.33% vs 46.67%, χ^2^ = 31.11, *P* < 0.001). The optimized jugular vein catheterization could make the SA model more stable, reliable and efficient than the traditional operation. Compared with traditional methods, our optimized scheme made numerous improvements in materials and techniques including uniformity, individualized variability of the S-type positioning nail, the length and connection matching, the shape of the end and low cost. Our optimized scheme could provide a more stable and efficient tool for basic research on drug addiction. Several subtle improvements under our personal experience are usually important for augmenting operational efficiency.

## Introduction

Studies on the mechanisms underlying the occurrence and development of most human diseases are usually based on good and efficient animal models. Therefore, good animal models are an important basis for scientific research, especially basic research.

Drug addiction is a progressive and chronic recurrent brain disease, characterized by an early stage of voluntary or recreational drug use followed by a stage of relatively regular drug use, then compulsive drug use and seeking, and finally, a loss of control over intake^1–3^. Classic animal models for drug addiction mainly include the Conditional Position Preference (CPP) model^4^ and the Self-administration (SA) model^5^. The operation to create the CPP model is relatively simple and convenient, but the individual differences among the experimental animals are too large, which leads to poor stability and reliability for drug addiction behaviors. In the SA model, the individual differences among experimental animals are relatively small, and it can provide the most direct point-to-point correspondence with addictive behavior that occurs in the natural environment, so it is highly reliable and exhibits both face validity and species generality^6^. Traditionally, the classical SA model has been the most effective method for studying drug addiction in laboratory animals.

However, the operation required to create the SA model is relatively complicated, and the technical requirements are rather steep. Indeed, SA techniques are usually limited by the fact that intravenous catheters need to last at least several weeks in the animal. In the SA model for freely-moving rats, the difficulty lies in ensuring that there is no blockage, leakage, infection and other problems in jugular vein catheterization, and all the information needs to be taught well during the whole training process. Therefore, the most critical technique is the jugular vein catheterization during the construction of a SA model for drug addiction. Although many intravenous implanted catheters are commercially available^7^, they are usually too expensive, and their practicality may not be as good as homemade catheters for specialized purposes.

Traditional jugular vein catheterization has many drawbacks, and it is prone to a series of problems such as blockage, leaking, infection and even death^8^. Once the above problems occur, the jugular vein catheterization would fail, affecting the construction of the next animal model and either delaying the whole experiment or resulting in the eventual failure of the entire experiment. In response to the above problems, through deep learning and our personal optimized experience, we have made many improvements to the traditional jugular vein catheter materials and implantation techniques and explored the feasibility and effectiveness of our optimized scheme.

A scientific and efficient animal model is the basis for basic research. Although the SA model has been used for many years, it should be noted that our personal optimized experience is still very important for the development and improvement of related techniques. Technical innovations have vastly improved the feasibility of long-term studies in rodents. Subtle improvements are also important for augmenting operational efficiency. To a certain extent, efficient animal models could reduce wastes in, for example, manpower, material and financial resources, and further improve the efficiency of scientific research. In the present study, we provided numerous details, including materials, approaches, updated techniques and protocols, for the development of a more optimal SA model. Our experiment aimed to optimize traditional methods and improve the success rate of animal jugular vein catheterization, as well as to provide a more stable and efficient scheme for developing a SA model for drug addiction.

## Materials and methods

### Animals

All animal procedures in our experiments were consistent with the guidelines of the Committee for Animal Care and Use (No. TDLL2020-12-152, Tangdu Hospital, Air Force Medical University, Xi’an, Shaanxi, China). And the experimental protocols were approved by the Committee for Animal Care and Use of Tangdu Hospital, Air Force Medical University. This study is in accordance with ARRIVE guidelines. Sprague–Dawley rats (Male; 300–350 g) were individually housed in our animal center under controlled temperature (22 ± 2 °C) and humidity (40–60%) on a 12-h light–dark cycle (lights on at 7:00 a.m.), and they were provided with food and water ad libitum. To adapt to the environment, we’ll keep healthy Sprague–Dawley rats (Male; 250–280 g) in our lab for at least a week, and they are more suitable for our following experiment.

### Optimized jugular vein catheter

**Figure 1:**
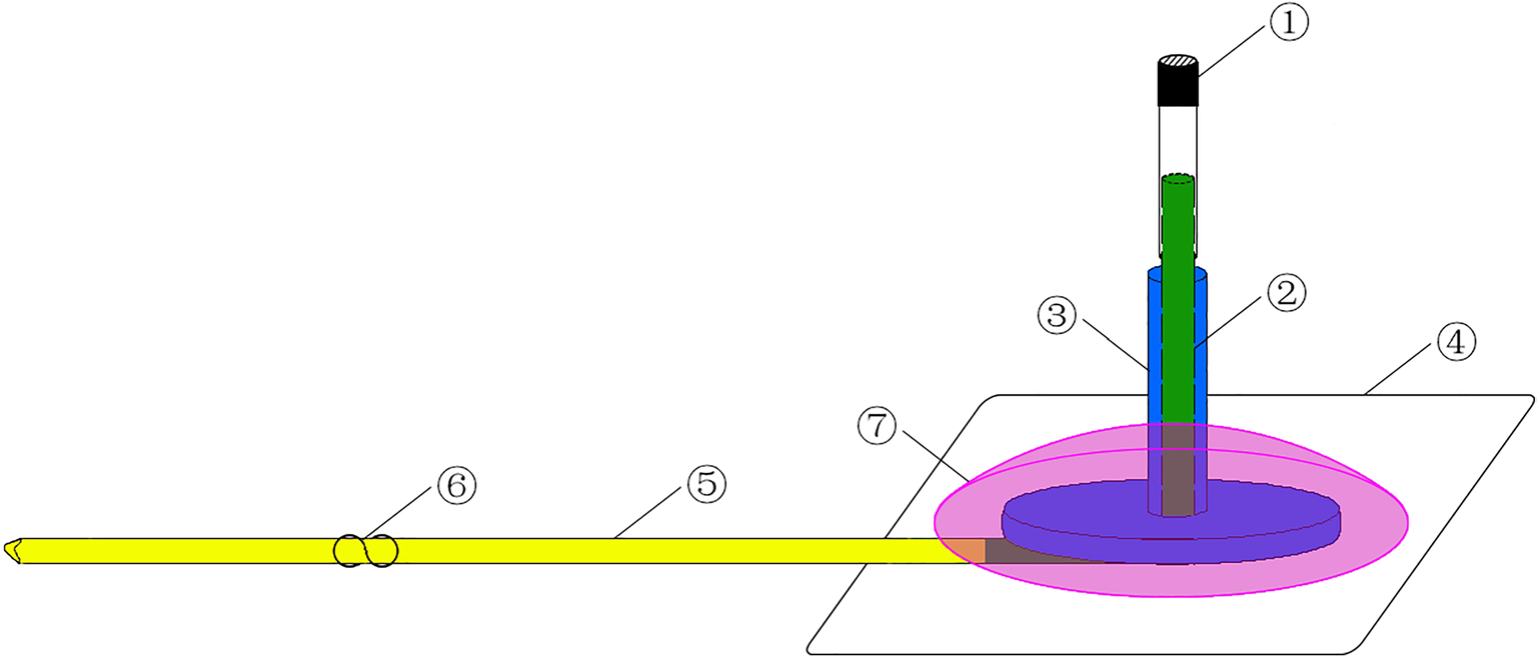
The schematic map of optimized jugular vein catheter. ① a PE tube cap; ② an L-shaped stainless steel tube; ③ an M4-style threaded base with inner hole; ④ a Nylon mesh; ⑤ a silicone tube; ⑥ an S-shaped positioning nail; ⑦ Hot melt adhesives.

This optimized system mainly consisted of an M4-style threaded base with an inner hole (O.D. = 3 mm; I.D. = 1 mm; Length = 18 mm), an L-shaped stainless steel tube (O.D. = 0.60 mm; I.D. = 0.30 mm; Length^1^ = 5 mm; Length^2^ = 20 mm), a nylon mesh (Φ = 20 mm), a silicone tube (O.D. = 0.90 mm; I.D. = 0.50 mm; Length = 95 mm), an S-shaped positioning nail and a PE tube cap (O.D. = 0.90 mm; I.D. = 0.58 mm; Length = 10 mm). The S-type positioning nail is made from a common stapler nail. We can adjust the position of the fixed site according to our actual needs by sliding it along the silicone tube. The connections of each component are shown in Fig. 1.

### Optimized jugular vein catheterization procedure

**Figure 2:**
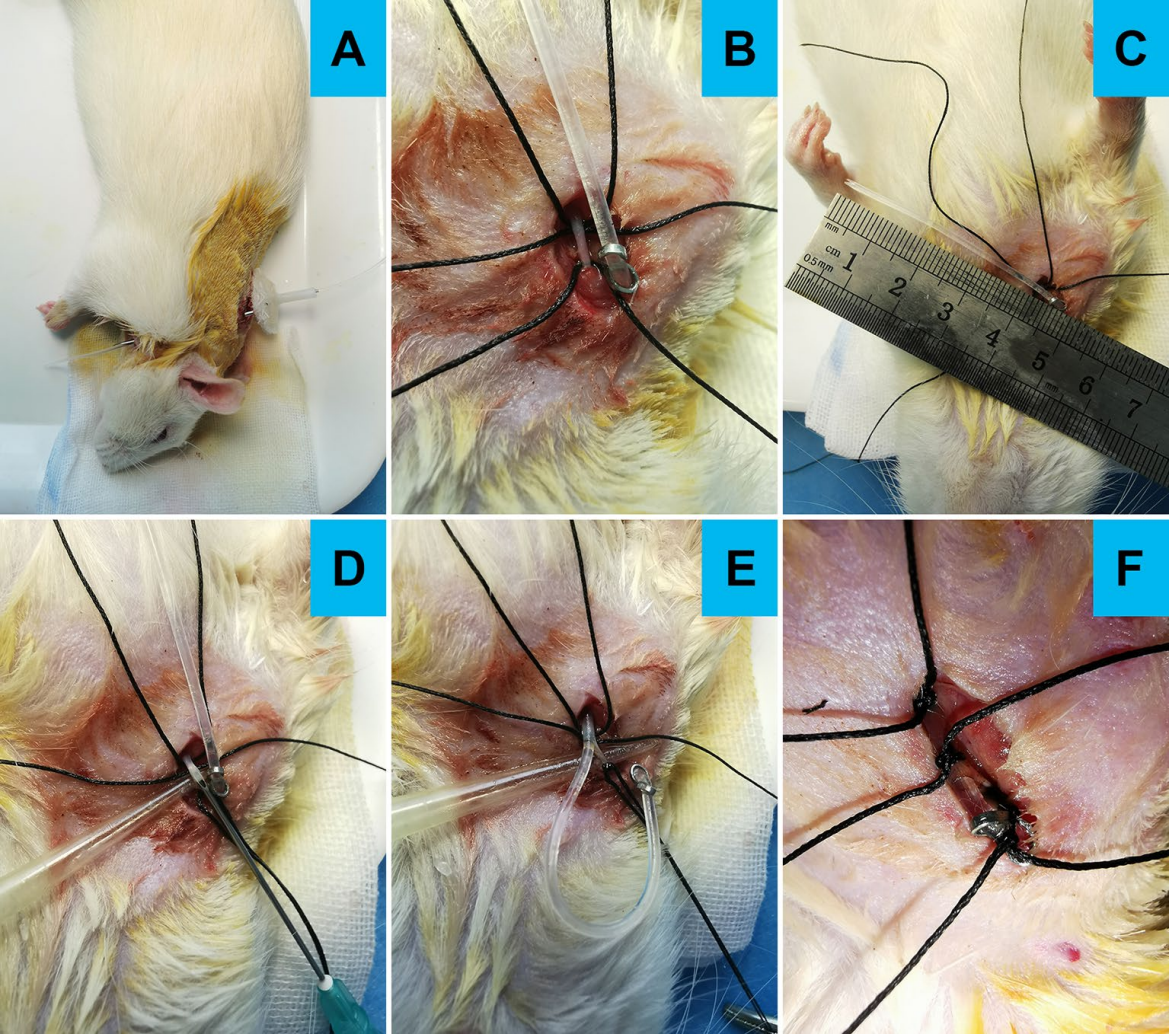
Several details of optimized jugular vein catheterization procedure. (**A**) The jugular vein catheter was inserted into the neck incision from the back incision through the subcutaneous tunnel. (**B**) Adjust and fix the S-shaped positioning nail. (**C**) Cut the silicone tube and trim into the shape of a fish mouth. (**D**) A syringe needle pierced the right jugular vein longitudinally. (**E**) The sterile silicone catheter was inserted into the jugular vein. (**F**) Fix the catheter and the proximal vessel.

Rats were anesthetized with sodium pentobarbital (50 mg/kg body weight, i.p.) and fixed in the supine position. A skin opening approximately 0.8 cm in length was cut longitudinally in the right neck to fully expose the right jugular vein. Similarly, a skin opening approximately 1.0 cm in length was cut longitudinally in the scapular region, and subcutaneous tissue was appropriately separated. Through a hypodermic tunnel needle, the optimized jugular vein catheter was inserted into the neck incision from the back incision. We set the S-shaped positioning nail on the silicone tube and adjusted its position so that the distance between the positioning nail and the end of the silicone tube was approximately 4.50 cm. Then, the end of the silicone tube was trimmed into the shape of a fish mouth. Three sutures were traversed under the right jugular vein, one of which was ligated to the distal end of the right jugular vein. After a syringe needle pierced the right jugular vein longitudinally, the sterile silicone catheter was inserted. The other two sutures were used to fix the catheter and the proximal vessel. Then, the positioning nail was secured together with the subcutaneous tissue to prevent the silicone tube from slipping out. Finally, we sutured the skin after disinfecting it. Following surgery, the jugular vein catheter was rinsed daily with saline containing heparin (10 U/mL) and penicillin (200, 000 IU/rat) to prevent catheter blockage and infection, respectively (Fig. 2 and Video 1).

### Drug self-administration apparatus

The self-administration (SA) apparatus (40 cm × 40 cm × 50 cm) was made in AniLab Co., Ltd., Ningbo, Zhejiang, China. The system could receive an input signal once the rat touched a valid nose-poke hole and produce a variety of output signals per our requirements (for example, pump, signal lights, nose-poke lights, or sounds). The top of the apparatus is a lever with a delivery tube and a shaft connection. To protect it, a spring tube comes out of the delivery tube. The upper end of the tube is connected to an injection pump through a liquid phase shaft, and the lower end is connected to the jugular vein catheter. In addition, a camera on top of the box can make recordings during the training procedure.

### Drug preparation

Cocaine hydrochloride (Qinghai Pharmaceutical Co. Ltd., Xining, Qinghai, China), dissolved in saline to a final concentration of 5 mg/mL, was stored at room temperature away from light.

### Drug self-administration training procedure

1. According to our experimental requirements, we first needed to create an appropriate experimental method (Fixed ratio, FR = 1). Specifically, when the experimental rats finish a valid nose-poke, the system pumps the drug once (T = 1.25 s, ν = 1.60 mL/min), which accompanies a series of changes in environmental cues such as signal lights (a green light turning off and a red light turning on for 20 s), a valid nose-poke light (T = 20 s), and a sound (buzzer, T = 1.25 s). When a valid nose-poke is completed, the system immediately enters a 20-s refractory period. During this refractory period, the system produces no output signals for any nose-poke behavior. After the 20-s refractory period, the system completes the first cycle and then enters the next cycle (the red light turns off and the green light turns on until the next valid nose-poke). Additionally, the system also produces no output signal when the experimental rat completes a control nose-poke.
2. After 7 days of recovery following the jugular vein catheterization operation, all rats underwent a drug self-administration training procedure over a 14-day training schedule. In the Cocaine group, when a rat finishes a valid nose-poke, the system pumps cocaine once via its right jugular vein, accompanied by several changes in the above environmental cues. In the Saline group, the system pumps an equal dose of saline once with the same changes in the above environmental cues. The training last 2 h every day, and the number of nose-pokes (left nose-poke and right nose-poke) and the number of pumps were recorded.

### Statistical analysis

All results in this experiment were expressed as the means ± SEMs. The data were statistically assessed via Student’s two-tailed t-test, one-way ANOVA, and Fisher’s exact test using SPSS 18.0 (SPSS Inc., USA). *P* values less than 0.05 were considered statistically significant. All group data in our study are reported in the table or figure legends.

## Results

### Jugular vein catheterization

**Figure 3:**
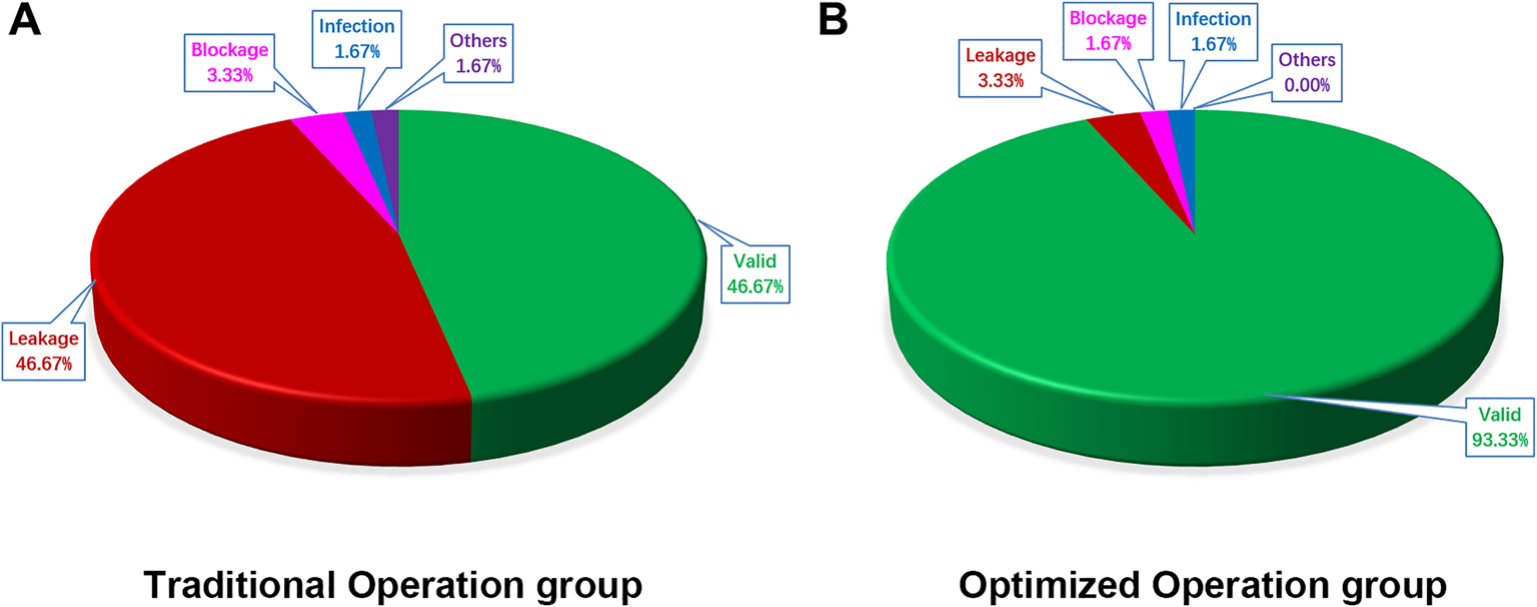
The effect of jugular vein catheterization for rats in two group. The statistical results showed the success rate of operation in the Traditional Operation group (TO group) was 46.67% and that in the Optimized Operation group (OO group) was 93.33%.

The optimized jugular vein catheterization procedure was described in the “Materials and methods” section above (Fig. 2 and Video 1). As shown in Table 1, surgical complications for jugular vein catheterization mainly included blockage, leakage, infection, and even death. Among them, the most frequent complication was leakage. A comprehensive analysis of our results showed that the success rate of the operation in the Traditional Operation group (TO group) was 46.67% and that in the Optimized Operation group (OO group) was 93.33% (Table 1). Fisher’s exact test in the TO group and OO group showed that the difference was very significant (χ^2^ = 31.11, *P* < 0.001) (Table 2), which indicated that the optimized jugular vein catheterization for rats was significantly better than the traditional operation. Figure 3 showed that statistical charts for the effect of jugular vein catheterization in the TO group and OO group.

**Table 1:** Statistical results of the effect of jugular vein catheterization for rats in the Traditional Operation group and the Optimized Operation group.

Jugular vein catheterization grouping	TO group		OO group	
Self-administration training grouping	ST group	CT group	ST group	CT group
**Surgery complications**				
Blockage	1	1	0	1
Leakage	13	15	1	1
Infection	0	1	1	0
Others	1	0	0	0
Valid	15	13	28	28
Total	30	30	30	30

*TO group* Traditional operation group, *OO group* Optimized operation group, *ST group* Saline training group, *CT group* Cocaine training group.

**Table 2:** Statistical results of Fisher’s exact test in in traditional operation group and the optimized operation group.

	Valid	Invalid	Total	Success rate (%)
TO group	28	32	60	46.67
OO group	56	4	60	93.33
Total	84	36	120	

*TO group* Traditional operation group, *OO group* Optimized operation group.

### Drug self-administration behavior in experimental rats

**Figure 4:**
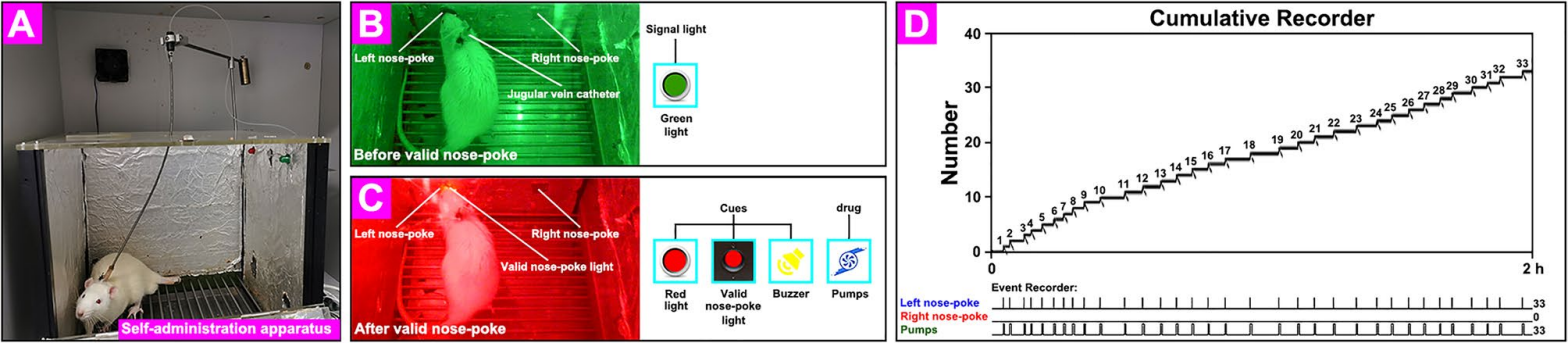
Self-administration training for rats. (**A**) Self-administration apparatus. (**B**, **C**) The comparison before and after valid nose-poke for experimental rat. Before valid nose-poke, only green light is on (B). When the experimental rats finish a valid nose-poke, the system pumps the drug once (T = 1.25 s, ν = 1.60 mL/min), which accompanies a series of changes in environmental cues such as signal lights (a green light turning off and a red light turning on for 20 s), a valid nose-poke light (T = 20 s), and a sound (buzzer, T = 1.25 s) (**C**). (**D**) The original recording of experimental rat’s behavioral events.

In the SA model, when the experimental rat performed a valid nose-poke, cocaine was pumped via the jugular vein catheter, which was accompanied by a series of changes in environmental cues such as lights and sounds. The rats were trained for 2 h every day, and the number of nose-pokes (left nose-poke and right nose-poke) and the number of pumps were recorded (Fig. 4 and Video 2). The addictive effect in the experimental rats in the Cocaine Training group was basically stable after approximately two weeks of training.

In this experiment, the results observed for the experimental rat self-administration behaviors were as follows:

#### Traditional operation group (TO group)

**Figure 5:**
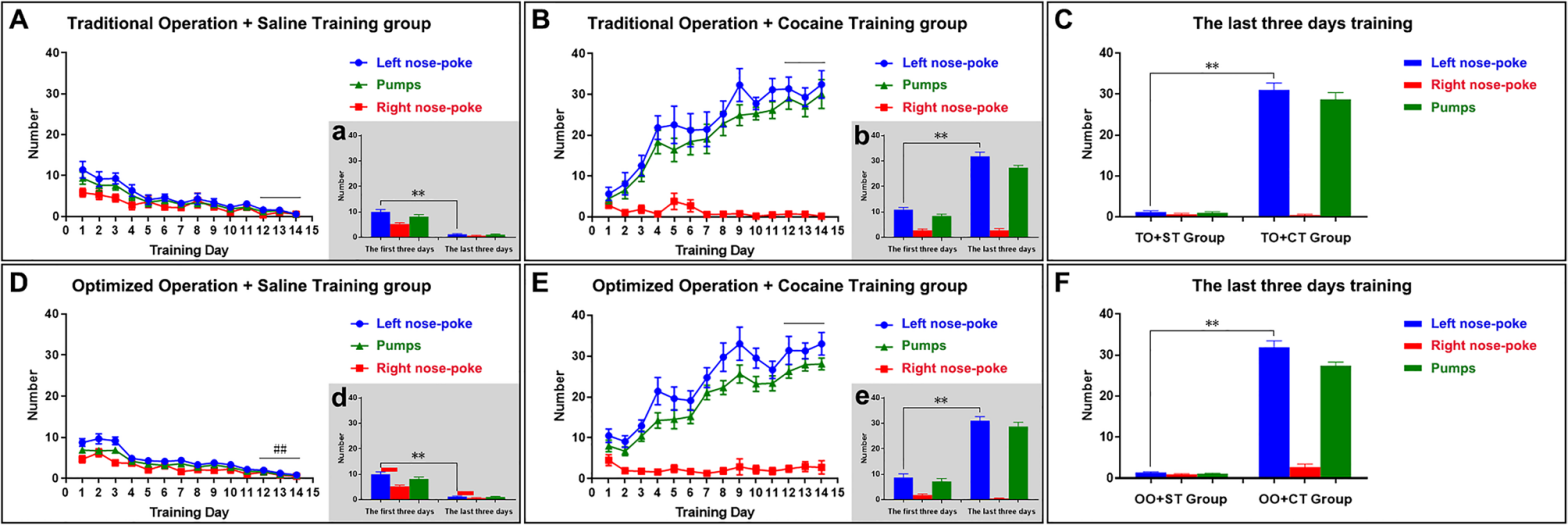
Effective establishment of cocaine self-administration model for rats. (**A**, **B**, **D**, **E**) The cocaine self-administration training everyday. (**A**) Traditional Operation + Saline Training group (n = 15): Compared to the first three days, the number of valid nose-poke for the last three days decreased [Day_1–3_: 9.9333 ± 1.002, Day_12–14_: 1.222 ± 0.2480, t_TO+ST group_ = 8.243, *P* < 0.01 (a)] during saline SA training period and reached a relatively stable level during the last three days of training period (F_TO+ST group_ = 2.587, P = 0.1180). (**B**) Traditional Operation + Cocaine Training group (n = 13): Compared to the first three days, the number of valid nose-poke for the last three days increased significantly [Day_1–3_: 8.744 ± 1.390, Day_12–14_: 31.05 ± 1.6272, t_TO+CT group_ = 11.42, *P* < 0.01 (b)] during cocaine SA training period and kept relatively stable during the last three days of training period (F_TO+CT group_ = 2.616, P = 0.0946). (**C**) The number of valid nose-poke for the last three days between TO + CT group and TO + ST group was significant difference (Day_12–14_ in TO + ST group: 1.222 ± 0.2480, Day_12–14_ in TO + CT group: 31.05 ± 1.6272, t_TO+CT group, TO+ST group_ = 19.41, *P* < 0.01). (**D**) Optimized Operation + Saline Training group (n = 28): Compared to the first three days, the number of valid nose-poke for the last three days decreased [Day_1–3_: 9.1786 ± 0.5814, Day_12–14_: 1.381 ± 0.1561, t_OO+ST group_ = 13.05, *P* < 0.01 (d)] during saline SA training period and reached a relatively stable level during the last three days of training period (F_OO+ST group_ = 6.146, *P* < 0.01). (**E**) Optimized Operation + Cocaine Training group (n = 28): Compared to the first three days, the number of valid nose-poke for the last three days increased significantly [Day_1–3_: 10.83 ± 0.8865, Day_12–14_: 31.89 ± 1.5945, t_OO+CT group_ = 11.71, *P* < 0.01 (e)] during cocaine SA training period and kept relatively stable during the last three days of training period (F_OO+CT group_ = 0.2034, P = 0.7714). F. The number of valid nose-poke for the last three days between OO + CT group and OO + ST group was significant difference (Day_12–14_ in OO + ST group: 1.381 ± 0.1561, Day_12–14_ in OO + CT group: 31.89 ± 1.5945, t_OO+CT group, OO+ST group_ = 19.04, *P* < 0.01). *TO + ST group* Traditional Operation + Saline Training group, *TO + CT group* Traditional Operation + Cocaine Training group, *OO + ST group* Optimized Operation + Saline Training group, *OO + CT group* Optimized Operation + Cocaine Training group. Data were presented as means ± SEM, **P < 0.01.

1. Traditional Operation + Saline Training group (TO + ST group, n = 15): Compared to the first three days, the number of valid nose-pokes for the last three days significantly decreased (Day_1–3_: 9.9333 ± 1.002, Day_12–14_: 1.222 ± 0.2480, t_TO+ST group_ = 8.243, *P* < 0.01) during the saline SA training period and reached a relatively stable level during the last three days of the training period (F_TO+ST group_ = 2.587, *P* = 0.1180) (Fig. 5A, a).
2. Traditional Operation + Cocaine Training group (TO + CT group, n = 13): Compared to the first three days, the number of valid nose-pokes for the last three days increased significantly (Day_1–3_: 8.744 ± 1.390, Day_12–14_: 31.05 ± 1.6272, t_TO+CT group_ = 11.42, *P* < 0.01) during the cocaine SA training period and remained relatively stable during the last three days of the training period (F_TO+CT group_ = 2.616, *P* = 0.0946) (Fig. 5B, b).

The number of valid nose-pokes for the last three days between the TO + CT group and TO + ST group was significantly different (Day_12–14_ in TO + ST group: 1.222 ± 0.2480, Day_12–14_ in TO + CT group: 31.05 ± 1.6272, t_TO+CT group, TO+ST group_ = 19.41, *P* < 0.01). The above results showed that we effectively established of a cocaine self-administration model in the rats in the TO group (Fig. 5C).

#### Optimized operation group (OO group)

1. Optimized Operation + Saline Training group (OO + ST group, n = 28): Compared to the first three days, the number of valid nose-pokes for the last three days decreased significantly (Day_1–3_: 9.1786 ± 0.5814, Day_12–14_: 1.381 ± 0.1561, t_OO+ST group_ = 13.05, *P* < 0.01) during the saline SA training period and reached a relatively stable level during the last three days of the training period (F_OO+ST group_ = 6.146, *P* < 0.01) (Fig. 5D, d).
2. Optimized Operation + Cocaine Training group (OO + CT group, n = 28): Compared to the first three days, the number of valid nose-pokes for the last three days increased significantly (Day_1–3_: 10.83 ± 0.8865, Day_12–14_: 31.05 ± 31.89 ± 1.5945, t_OO+CT group_ = 11.71, *P* < 0.01) during the cocaine SA training period and remained relatively stable during the last three days of the training period (F_OO+CT group_ = 0.2034, *P* = 0.7714) (Fig. 5E, e).

The number of valid nose-pokes for the last three days between the OO + CT group and the OO + ST group was significantly different (Day_12–14_ in OO + ST group: 1.381 ± 0.1561, Day_12–14_ in OO + CT group: 31.89 ± 1.5945, t_OO+CT group, OO+ST group_ = 19.04, *P* < 0.01). These results showed that we also effectively established a cocaine self-administration model in the rats in the OO group (Fig. 5F).

In total, from the results observed for the drug self-administration behavior, although the cocaine self-administration model could be effectively established in the experimental rats in both the TO group and OO group, the number of successful samples in the two groups was significantly different. The number of successful samples in the OO group was much greater than that in the TO group. The optimized jugular vein catheterization could make the construction of a drug self-administration model in rats more stable and reliable and was more efficient in successfully creating SA models than the traditional operation.

## Discussion

We observed and analyzed the success rate of two kinds of jugular vein catheterizations in a drug self-administration (SA) rat model. The success rate of the jugular vein catheterization in the Optimized Operation group (OO group) was significantly higher than that in the Traditional Operation group (TO group) (93.33% vs 46.67%, χ^2^ = 31.11, *P* < 0.001). Thus, the optimized jugular vein catheterization could make the construction of a drug self-administration model in rats more stable and reliable and was more efficient in successfully creating SA models than the traditional operation.

A good animal model serves as the basis for the study of specific mechanisms for the occurrence and development of related diseases, and it is also an important tool for scientific research, especially basic research. In animal experiments on drug addiction, a large number of studies have shown that compared with the CPP model, the SA model is more stable and more reliable, results in fewer individual differences and is considered to be one of the most effective models for studying animal drug-craving behavior^9^. Animals used to create SA models for drug addiction mainly include rats, mice and nonhuman primates.

In the middle of the twentieth century, many researchers began to perform drug-related behavioral studies with animals to better understand the human addiction process. Professor James R. Weeks used automatic intravenous injection to simulate morphine addiction in non-free-living rats in 1962^10^. This signified the start of studies using SA models with jugular vein catheterization. The industry has adopted its own drug delivery model as a behavioral method for studying drug addiction^9^. In 1969, Professors Deneau, Yanagita, and Seevers conducted a study on drug abuse for free-living cynomolgus monkeys to explore whether they would perform voluntary and active self-administration, which was much closer to the state observed in human addiction^11^.

Under the SA training procedure, when the experimental animal performs a response, such as performing a valid nose-poke or pressing a valid lever, a certain dose of drug is delivered via an intravenous catheter. However, the typical operation for creating the SA model is relatively complicated, and its technical requirements are steep. In a SA model in free-moving rats, it is most important to ensure that the jugular vein is unobstructed, not leaking, and is free of infection and other problems during the whole training process. The most challenging and critical technique is the jugular vein catheterization^12^.

The traditional tube operation primarily includes a PE tube (the extravascular part), a silicone tube placed into the blood vessel, and a fixing point created by creating a knot at the connection between the two sections. However, due to the instability of the structure itself, the possibility of leaking at the joint is high^13,14^. This method has many drawbacks, and it is prone to problems such as blockage, leakage, bleeding, infection, significant weight loss, and even death. If any of the above problems occurs, the jugular vein catheterization would fail, directly and negatively impacting the construction of additional animal models^15^.

There are two main reasons for the low success rate of the traditional jugular vein catheterization surgery: (1) The components of the implanted catheter are complex in structure, the flexibility and strength are not suitable for long-term implantation, and the two components are not perfectly matched, easily resulting in blockage or leakage; (2) the surgical operation is not standard or rigorous, resulting in an unsuccessful implantation.

In response to the above problems, our experiment made several following improvements in the materials and techniques used for performing jugular vein catheterization to explore the feasibility and effectiveness of a more optimized scheme:

1. *Uniformity* The entire tube is composed of soft silicone, which eliminates the need for a secondary joint. The possibility of leakage is also decreased, and the soft silicone tube has little effect on the neck flexibility of the rats. The traditional catheter, however, uses a PE tube (the extravascular part) and a silicone tube (placed in the blood vessel), which may increase the possibility of leakage at the joint.
2. *Individualized variability of the S-type positioning nail* the S-type positioning nail is made from a common stapler nail. In our opinion, this is the most important and satisfying improvement, because we could adjust the position of the fixed site according to our actual needs after adding the small S-type positioning nail. The traditional method uses one knot directly at the connection between the two parts. The stability of this knot is not as good as a solid knot in a rope. If the knot is too tight, the catheter could be easily blocked. If it is too loose, the tube could easily be disengaged or affect the flexibility of the neck. The most serious consequence would be dragging of the silicone tube placed in the vein. In addition, the proposed operation might be relatively easy because of the use of the S-type positioning nail, which could improve the surgical efficiency.
3. *The length matching of the implanted catheter* it seems impossible to prepare precise implanted catheters in advance due to individual differences in the experimental rats. In our optimized materials, because of the S-type positioning nail, both the length of the catheter and the position of the fixed site could be adjusted according to our actual needs. However, the traditional materials cannot be freely adjusted due to the existence of a uniform fixed position, so it is impossible to match the implantation perfectly for each rat.
4. *The implanted blood vessel portion of the catheter* The length of the implanted blood vessel portion of the optimized catheter is approximately 4.5 cm. At this length, the end of the catheter just reaches the right atrium, which can reduce direct damage to blood vessels. To some extent, the length of the implanted portion also reduces the risk of blockage and leakage. However, in the traditional procedure, the implanted blood vessel portion is generally 1.2 cm in length, increasing the chance of damaging blood vessels and leading to thrombi and blockage.
5. *The shape of the end of the implanted catheter* In our procedure, the end is cut into the shape of a fish mouth shape so that the implanted catheter cannot easily adhere to the vessel wall and damage it. However, in the traditional procedure, the end of the implanted catheter has a single inclined surface (similar to a syringe needle), making it easy to damage the blood vessels and ultimately resulting in leakage.
6. *The connection between the jugular vein catheter and the drug self-administration device* In our procedure, an M4 threaded base with an internal bore is used to connect the jugular vein catheter to the drug self-administration device. The M4 threaded base is small, the base is completely embedded in the back of the experimental rat, and the upper thread is only partially exposed. The connection between the upper thread portion and the drug self-administration device is stable and cannot easily fall off, and it does not affect the flexibility of the rats. In other studies, experimental rats needed a small back clip to connect the jugular vein catheter to the drug self-administration device. However, free-moving rats sometimes easily shake off the clip because it makes them uncomfortable. Furthermore, in the traditional operation, it is easier to drag the jugular vein catheter, which eventually leads to failure of the model ^15^.
7. *Low cost of materials* the optimized jugular vein catheters are mainly composed of several simple and inexpensive components. It should be emphasized that simplicity, low cost and high efficiency have been effectively unified due to the application of the S-type positioning nail.

The success of the jugular vein catheterization is the most important factor for the SA model. Our results showed that the optimized jugular vein catheterization for rats was significantly better than the traditional procedure. The success rate of jugular vein catheterization was significantly improved after optimization.

In summary, the construction of scientific and efficient animal models is the basis for basic scientific research. The SA model represents one of the oldest methods for studying drug addiction, but it will continue to provide very valuable contributions to research on drug addiction well in the future. Jugular vein catheterization is one of the most important and critical techniques in this animal model. In the present study, we provided numerous details, including materials, approaches, updated techniques and protocols, for the development of a more optimal SA model. Our experiment explored an optimized scheme for animal jugular vein catheterization for the creation of a drug self-administration model, which could provide a more stable and efficient tool for basic research on drug addiction.

## Limitation

In our data, there was a little weakness: the drug self-administration training procedure lasted for 14 days only. This was decided by the animal drug self-administration model for drug addiction. A period of two weeks of drug self-administration is current standards for drug addiction. In general, a 14-day training schedule was enough and reasonable for the construction of this animal model, because the behavior of experimental rats was enough stable and reliable. The aim of technical innovations was to achieve maximum efficiency with the least resources, so this simple and efficient measure was a great progress in scientific research. Certainly, the self-administration training (e.g. medicines) could be extended appropriately for over one or two months or more. In fact, our another experiment lasted almost two months for another purpose, but not for this basic self-administration model for drug addiction. I meant that a period of two weeks was not the upper limit of this technology. We just took two weeks of data for analysis in this experiment (the fundamental construction of experimental rats drug self-administration model for drug addiction such as cocaine, heroin, morphine). The lower success rate of the jugular vein catheterization surgery for the self-administration model for drug addiction was caused by the effects of addictive drugs (e.g. cocaine, heroin, morphine, etc.), such as animal mania, restlessness, excitement.

## Supplementary Information

Supplementary Video 1.

Supplementary Video 2.

## Abbreviations

SA: Self-administration
CPP: Conditioned place preference
i.p.: Intraperitoneal injection
O.D.: Outside diameter
I.D.: Inside diameter
